# The androgen receptor cytosine-adenine-guanine repeat length contributes to the development of epithelial ovarian cancer

**DOI:** 10.18632/oncotarget.6012

**Published:** 2015-10-20

**Authors:** Xiangrui Meng, Peng Lu, Zhi Chu, Qingxia Fan

**Affiliations:** ^1^ Department of Oncology, The First Affiliated Hospital of Zhengzhou University, Zheng Zhou, People's Republic of China; ^2^ Department of Gastrointestinal Surgery, People's Hospital of Zhengzhou, Zheng Zhou, People's Republic of China; ^3^ General Hospital, Jinan Military Command, Jinan, Shandong Province, People's Republic of China

**Keywords:** ovarian cancer, AR, CAG, repeat, polymorphism

## Abstract

Ovarian cancer is the main cause of death among women with gynecological malignancies. Androgen and its receptors play an important role in ovarian cancer pathogenesis. Here, We aim to evaluate the relationship between *AR* CAG and GGN repeat length polymorphisms and Epithelial Ovarian Cancer (EOC) risk in a two-stage, case-control study among Chinese women. The repeat length was analyzed as a categorical variable for CAG_A and GGN_A (average allele), CAG-S and GGN_S (shorter allele), CAG-L and GGN_L (longer allele), respectively. The median value of the repeat length among the controls was used as the cutoff point. Women with longer AR CAG repeats had a decreased risk of developing EOC. The results was replicated in an independent samples. Compared to those with shorter (<22) CAG_A repeat length, women with longer (≥22) CAG_A repeats length had a 31% decreased EOC risk (OR = 0.69, 95% CI: 0.62–0.77, *P* = 5.06 × 10^−11^). For CAG_S and CAG_L, the results remain consistent. However, we didn't detected any significant associations for GGN_A, GGN_S, and GGN_L. This should be the first study to examine the association between *AR* repeat length polymorphisms and ovarian cancer risk in a relatively large group of Asian women.

## INTRODUCTION

According to American Cancer Society's report, ovarian cancer ranks 5th overall for cancer death in women, accounting for 5% of all cancer deaths in women [1]. Although previous studies have demonstrated that some etiologic factors, including early age at menarche, late age at menopause, obesity, use of estrogen and hormone-replacement therapy, and inherited susceptibility, are positively associated with ovarian cancer, however, the common genetic variants explain less than 3.1% of the excess familial risk of EOC so additional susceptibility loci are likely to exist [2–5].

Epidemiologic and biological data suggest a role for androgens and androgen receptor (AR) in ovarian cancer development [6, 7]. The *AR* is a nuclear transcription factor that mediates the actions of testosterone and dihydrotestosterone [8]. Since the early studies with tritiated DHT exchange assays carried out by the McGuire's laboratory in the seventies, the presence of *AR* protein in cancer specimens has been detected [9]. In particular, two microsatellite polymorphic variants (CAG)n and (GGN)n repeats were found in exon 1 of the *AR* gene [8, 10]. Together, these polymorphisms make 90% of women heterocygotic for the *AR* gene. The CAG trinucleotide repeat codes for a polyglutamine tract which normally ranges from 6 to 39 repeats [11]. Molecular analyses have shown that the transactivation capacity of the *AR* decreases with increasing number of glutamines encoded by the CAG repeat tract [12]. The biological effects of changing the GGN repeat length have not been as widely studied as those of CAG. Studies found that *AR* protein levels were inversely affected by the GGN repeat length due to the reason of the GGN repeat forming a hairpin structure in *AR* mRNA [13].

There are several published studies that addressed the association between *AR* repeat polymorphisms and ovarian cancer [6, 12, 14–17]. However, none was conducted in non-Asians. Considering the important role of *AR* repeat polymorphisms in the carcinogenic process, we carried out a two-stage case-control study in a Chinese population to investigate the possible relationship between these two *AR* repeat polymorphisms and the risk of Epithelial ovarian cancer (EOC) in the Chinese population.

## RESULTS

**Figure 1:**
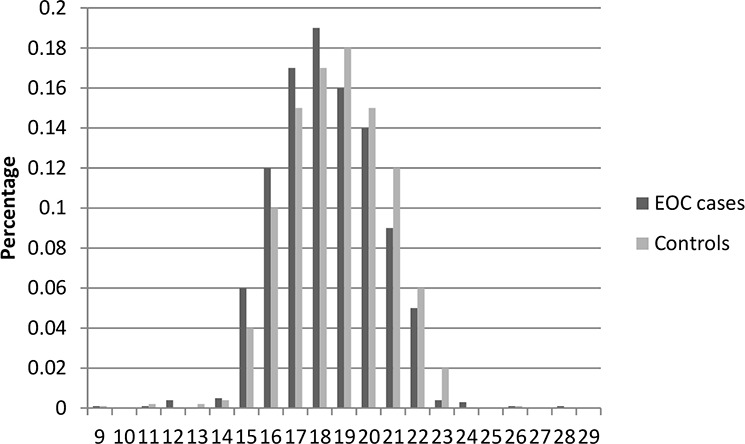
Distribution of AR CAG repeat number for the shorter allele among EOC cases and controls in stage 1

**Figure 2:**
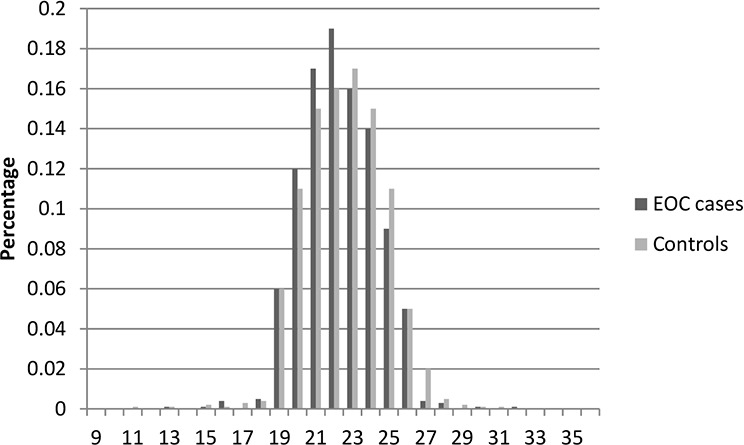
Distribution of AR CAG repeat number for the longer allele among EOC cases and controls in stage 1

**Figure 3:**
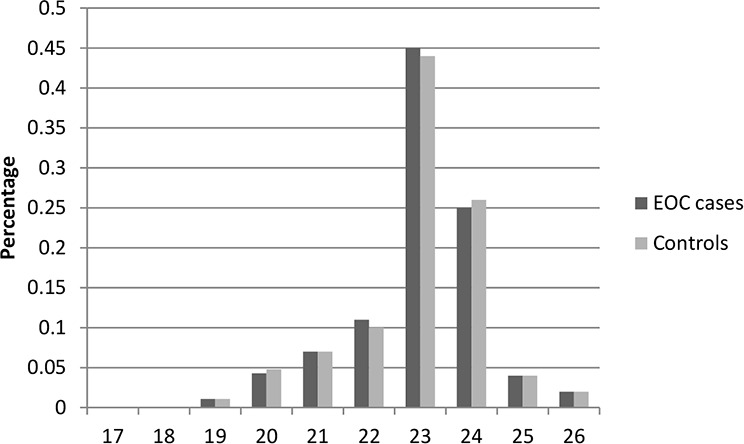
Distribution of AR GGN repeat number for the shorter allele among EOC cases and controls in stage 1

**Figure 4:**
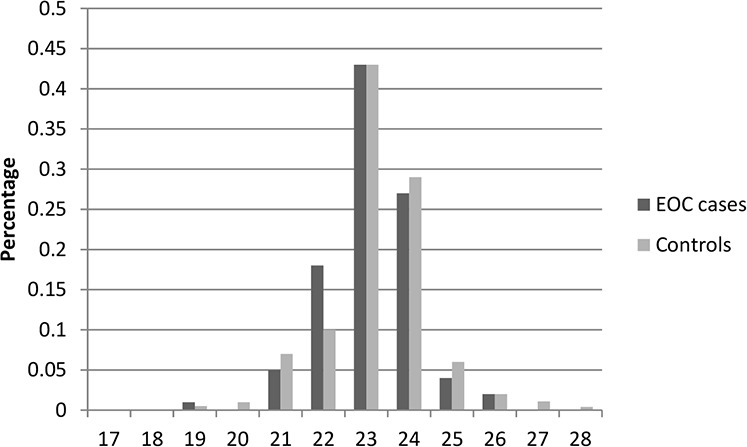
Distribution of AR GGN repeat number for the longer allele among EOC cases and controls in stage 1

**Figure 5:**
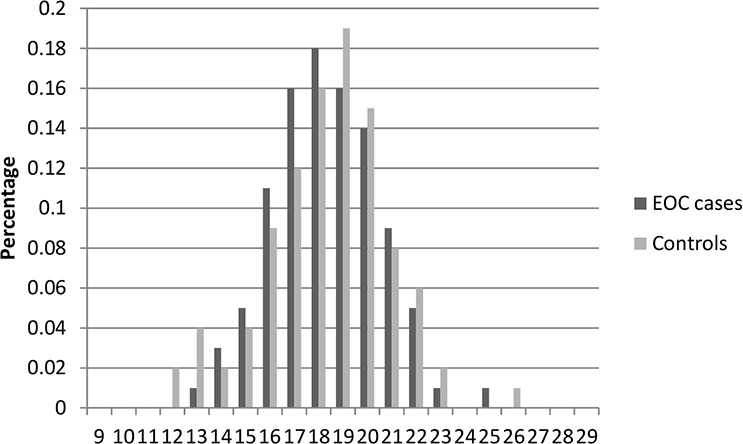
Distribution of AR CAG repeat number for the shorter allele among EOC cases and controls in stage 2

**Figure 6:**
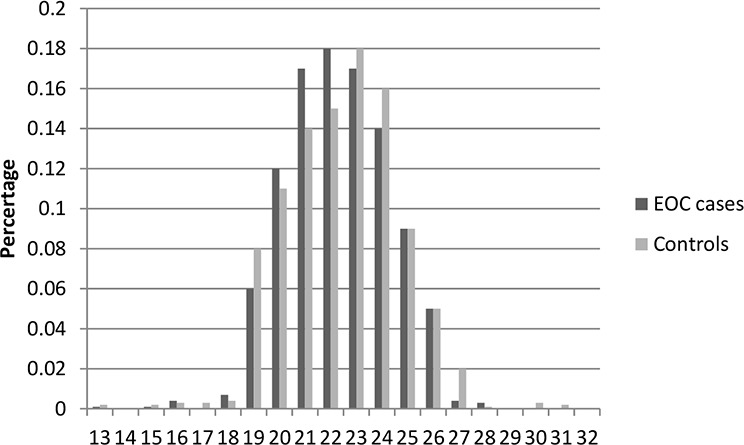
Distribution of AR CAG repeat number for the longer allele among EOC cases and controls in stage 2

Table 1 presents the characteristics of the study population included in this study. In total, there were 2,795 cases and 2,800 controls included in this two-stage study. No significant differences were observed between cases and controls in distribution of age, education, smoking, and body mass index (BMI) in both stages, indicating satisfactory matching for the case-control studies. We found that there were significant differences in usage of hormone replacement therapy between cases and controls (Stage1: *P* = 0.028; Stage1: *P* = 0.0009), which indicated that usage of hormone replacement therapy might be a potential risk factor for EOC risk. The most histological subtype was serous (71% in Stage 1 and 73% in Stage 2). Figures 1–6 showed the distribution of CAG and GGN repeat sequences between the control group and EOC patients, respectively. Range of allele length have been given for both the shorter and longer allele.

**Table 1:** Characteristics of EOC patients and healthy controls used in this study

Category	Stage 1			Stage 2		
	Cases (*N* = 1,925)	Controls (*N* = 1,900)	*P* Value	Cases (*N* = 870)	Controls (*N* = 900)	*P* Value
Age (yr)						
Range	35–74	34–72	-	33–78	33–75	-
Mean ± SD	51.0 ± 7.5	50.9 ± 7.1	0.672	53.7 ± 5.5	53.2 ± 5.3	0.052
Education						
less than middle school	764 (39.7%)	709 (37.3%)	0.132	371 (42.7%)	381 (42.3%)	0.895
middle school and above	1,161 (60.3%)	1,191 (62.7%)		499 (57.3%)	519 (57.7%)	
Ever smoker						
Yes	279 (14.5%)	268 (14.1%)	0.731	129 (14.8%)	132 (14.7%)	0.924
No	1,646 (85.5%)	1,632 (85.9%)		741 (85.2%)	768 (85.3%)	
Use of hormone replacement therapy						
Yes	81 (4.2%)	55 (2.9%)	**0.028**	43 (4.9%)	23 (2.5%)	**0.0009**
No	1,844 (95.8%)	1,845 (97.1%)		837 (95.1%)	877 (97.5%)	
Body mass index (kg/m2)	24.0 ± 3.7	23.8 ± 3.0	0.211	23.8 ± 3.1	23.6 ± 3.2	0.182
**Histopathology**						
Serous	1,367 (71%)			635 (73%)		
Mucinous	154 (8%)			78 (9%)		
Clear cell	116 (6%)			44 (5%)		
Endometrioid	192 (10%)			113 (13%)		
Others	96 (5%)			0 (0%)		

Table 2 presents the associations between *AR* CAG and GGN repeat length and EOC risk in stage 1. For CAG polymorphism, women in the category of longer (≥22) CAG_A repeats had a significant 34% decreased EOC risk (OR = 0.66, 95% CI: 0.57–0.75, *P* = 1.32 × 10^−9^), when compared to those with the shorter (<22) CAG_A repeat length. The results were consistent and also statistically significant for the CAG_L and CAG_S alleles. For GGN polymorphism, we didn't detected any significant associations for GGN_A, GGN_S, and GGN_L. Then association of *AR* CAG repeat length with EOC risk was validated in an independent stage 2 samples. As shown in Table 3, the significant associations for CAG_A, CAG_L and CAG_S were replicated. When combined together, women with longer (≥22) CAG_A repeats had a significant 31% decreased EOC risk (OR = 0.69, 95% CI: 0.62–0.77, *P* = 5.06 × 10^−11^), when compared to those with the shorter (<22).

**Table 2:** Association of AR repeat length with EOC risk in Stage 1

	Cases, *n* (%)	Controls, *n* (%)	OR (95% CI)*	*P*
**CAG repeat**				
CAG_A				
<22	728 (37.8%)	543 (28.6%)	Referent	
≥22	1197 (62.2%)	1357 (71.4%)	0.66 (0.57–0.75)	**1.32 × 10^−9^**
CAG_S				
<19	1061 (55.1%)	891 (46.9%)	Referent	
≥19	864 (44.9%)	1009 (53.1%)	0.72 (0.63–0.82)	**3.66 × 10^−7^**
CAG_L				
<23	1132 (58.8%)	935 (49.2%)	Referent	
≥23	793 (41.2%)	965 (50.8%)	0.68 (0.60–0.77)	**2.63 × 10^−9^**
				
**GGN repeat**				
GGN_A				
≤23	584 (67.1%)	578 (64.2%)	Referent	
>23	286 (32.9%)	322 (35.8%)	0.87 (0.72–1.07)	0.198
GGN _S				
≤23	595 (68.4%)	602 (66.9%)	Referent	
>23	275 (31.6%)	298 (33.1%)	0.93 (0.77–1.14)	0.500
GGN _L				
≤23	573 (65.9%)	554 (61.5%)	Referent	
>23	297 (34.1%)	346 (38.5%)	0.83 (0.68–1.01)	0.060

* adjusted for age, education and Use of hormone replacement therapy

**Table 3:** Association of AR repeat length with EOC risk in Stage 2 and combined results

	Cases, *n* (%)	Controls, *n* (%)	OR (95% CI)*	*P*
**CAG repeat**				
**Stage 2**				
CAG_A				
<22	320 (36.8%)	275 (30.6%)	Referent	
≥22	550 (63.2%)	625 (69.4%)	0.76 (0.62–0.92)	**0.006**
CAG_S				
<19	470 (54.0%)	441 (49.0%)	Referent	
≥19	400 (46.0%)	459 (51.0%)	0.82 (0.68–0.99)	**0.03**
CAG_L				
<23	480 (55.2%)	445 (49.4%)	Referent	
≥23	390 (44.8%)	455 (50.6%)	0.79 (0.66–0.96)	**0.01**
				
**Combined**				
CAG_A				
<22	1048 (37%)	818 (29%)	Referent	
≥22	1747 (63%)	1982 (71%)	0.69 (0.62–0.77)	**5.06 × 10^−11^**
CAG_S				
<19	1531 (55%)	1332 (48%)	Referent	
≥19	1264 (45%)	1468 (52%)	0.75 (0.67–0.83)	**7.02 × 10^−8^**
CAG_L				
<23	1612 (58%)	1380 (49%)	Referent	
≥23	1183 (42%)	1420 (51%)	0.71(0.64–0.79)	**3.18 × 10^−10^**

* adjusted for age, education and Use of hormone replacement therapy

## DISCUSSION

In this two-stage, case-control study, we examined the relationship between *AR* CAG and GGN repeat polymorphisms and EOC risk in Chinese women. We identified that women with longer *AR* CAG repeats had a decreased risk of developing EOC. To our knowledge, this is the first study to examine the association between *AR* repeat length polymorphisms and ovarian cancer risk in a relatively large group of Asian women.

The *AR* is codified by the *AR* gene which is located on the X chromosome (q11.2–q12). Two length polymorphisms were located at the exon 1: a 9–39 CAG repeat (polyglutamine, polyQ) and a 14–27 GGN repeat (polyglycine, polyG). Together, these polymorphisms make 90% of women heterocygotic for the *AR* gene [13]. First, a role in cancer predisposition for *AR* repeat polymorphisms was suggested by inversed associations between prostate cancer risk and CAG repeat length within the AR transactivation domain in 1995 [18]. Then, several studies aimed to address the associations between *AR* repeat polymorphism and ovarian cancer risk previously, although the results were inconsistent [6, 12, 14, 19–25]. Spurdle1 et al [20] first showed no evidence for an association between ovarian cancer risk and the genotype defined by the CAG polymorphism, although they cannot exclude small effects, or threshold effects in a small subgroup. Then, Dagan et al [19] explore the association of *AR* CAG repeat length with risk of ovarian cancer in Jewish Israeli women who are BRCA1/2 mutation carriers, and null results were concluded due to the small sample size. Since, several studies published recently found shorter CAG repeat length could increase risk of ovarian cancer [12, 14, 24, 25], while two studies published previously showed inversed results [6, 23]. One possible reason is the ethnically diverse populations, for which potential lifestyle and cultural factors, as well as other genetic factors, that may modify risk. Another reason is the big difference of the sample sizes, which may result in false results. Also, observed associations in some studies were of marginal significance. Therefore, these results could potentially be due to chance. In current study, we identified that women with longer AR CAG repeats had a decreased risk of developing EOC, using a large sample size, two-stage design. This results was also supported by another study by Li et al [26], who reported that short androgen receptor allele length is a poor prognostic factor in epithelial ovarian carcinoma.

Strength of this study includes large sample size and the two-stage study design, which is more efficient on discovering potential risk factors. Limitations of our study, including potential biases and population generalizability, should also be taken attentions. Because the study population is primarily Chinese women and our analyses are restricted to Chinese, our results are not generalizable to other ethnicities. However, the homogeneity of our population is an advantage. Due to our case-control design, we must also consider the possibility of selection bias.

Our findings suggest that shorter CAG repeat length, modulates the actions of androgens in ovarian neoplasia and tumor growth. These data add to the growing body of evidence linking androgenicity to the pathogenesis and tumor biology of ovarian cancer. Functional studies characterizing hormone activity and response pathways may reveal specific mechanisms in which androgens function in ovarian cancer biology.

## MATERIALS AND METHODS

### Subjects

There were 1,925 EOC cases and 1,900 cancer-free controls included in Stage 1, and Stage 2 included an additional EOC 870 cases and 900 controls. All subjects were genetically unrelated ethnic Han Chinese. Controls were frequency-matched to the cases by age (the range of five years), and residential area. All cases were newly diagnosed according to International Classification of Diseases for Oncology (ICD-O) codes. All the controls were genetically unrelated with cases. Subjects with histories of cancer were excluded from the control group. We also obtained clinical information for all the cases. After informed consent was obtained, a questionnaire about lifestyle factors was completed by all the subjects through face-to-face interviews. After the interview, each subject provided 3–5 mL of venous blood. Approval for this study was obtained from the institutional review boards. Written informed consent was obtained from all participants or from the patients’ representatives.

### Genotyping

DNA from blood of patients and control specimens was extracted from white blood cell fractions using the Qiagen Blood Kit (Qiagen, Valencia, CA). Then DNA was PCR-amplified using fluorescently labeled primers (F: 5′-TCCAGAATCTGTTCCAGAGCGTGC-3′; R: 5′-GCTG TGAAGGTTGCTGTTCCTCAT-3′) for the *AR* CAG repeat polymorphism, while using the primers (F: 5′-CGGTTCT GG GTCACCCTC A-3′; R: 5′-TCACCATGCCGCCAG GGTA-3′) for *AR* GGN repeat polymorphism, using the conditions as follows: with an initial denaturation step at 96°C followed by 35 cycles of 1 min at 96°C, 45 s at 61°C, and 2 min at 72°C, and a final extension step for 5 min at 72°C. Fragments were then run on denaturing polyacrylamide gels on the Applied Biosystems Prism 3700XL and analyzed by Applied Biosystems Prism Genescan automated fluorescence detection (Applied Biosystems, Foster City, CA).

### Statistical analyses

We used the Chi-square test to examine the differences in the frequency distributions of categorical variables between groups. The Shapiro-Wilk test was used to verify the normality of distribution, and Levene's test for equality of variances. When Both of these assumptions were met, *t*-test was used to test the differences for the *AR* repeat lengths between case patients and control subjects. Otherwise, the Mann-Whitney *U* test was used. Odds ratios (ORs) and their 95% CI were calculated from unconditional logistic regression analyses. The repeat length was analyzed as a categorical variable for CAG_A and GGN_A (average allele), CAG-S and GGN_S (shorter allele), CAG-L and GGN_L (longer allele), respectively. The median value of the repeat length among the controls was used as the cutoff point. If some females have two alleles for the same repeat number, average allele, shorter allele, and longer allele share the same number without identifying which is longer. All statistical tests were 2-sided and a *P* value significance threshold of 0.05 was set. All analyses were conducted using SPSS 19.0.
